# Quercetin Reduces Lipid Accumulation During Adipogenic Differentiation in Association with Stage-Dependent Mitochondrial-Redox and Metabolic Alterations Revealed by Time-Resolved ^1^H NMR Metabolomics

**DOI:** 10.3390/life16091455

**Published:** 2026-08-31

**Authors:** Aye Thidar Moe Moe, Khin Thandar Htun, Jie Pan, Krit Jaikumkao, Nuttawadee Intachai, Anusorn Lungkaphin, Monruedee Tapanya, Duanghathai Pasanta, Chatchanok Udomtanakunchai, Montree Tungjai, Siriprapa Kaewjaeng, Hong Joo Kim, Jakrapong Kaewkhao, Christopher Lai, Suchart Kothan

**Affiliations:** 1 Center of Radiation Research and Medical Imaging, Department of Radiologic Technology, Faculty of Associated Medical Sciences, Chiang Mai University, Chiang Mai 50200, Thailand; ayethidar_moemoe@cmu.ac.th (A.T.M.M.); khinthandar_htun@cmu.ac.th (K.T.H.); krit.ja@cmu.ac.th (K.J.); nuttawadee.i@cmu.ac.th (N.I.); monruedee.t@cmu.ac.th (M.T.); duanghathai.pas@o365cmu.onmicrosoft.com (D.P.); chatchanok.u@cmu.ac.th (C.U.); montree.t@cmu.ac.th (M.T.); siriprapa.k@cmu.ac.th (S.K.); 2 Shandong Provincial Key Laboratory of Animal Resistant Biology, College of Life Sciences, Shandong Normal University, Jinan 250014, China; 3 Department of Physiology, Faculty of Medicine, Chiang Mai University, Chiang Mai 50200, Thailand; anusorn.lungka@cmu.ac.th; 4 Department of Physics, Kyungpook National University, Daegu 41566, Republic of Korea; hongjoo@knu.ac.kr; 5 Center of Excellence in Glass Technology and Materials Science (CEGM), Faculty of Science and Technology, Nakhon Pathom Rajabhat University, Nakhon Pathom 73000, Thailand; jakrapong@webmail.npru.ac.th; 6 School of Medical and Health Sciences, Tung Wah College, Hong Kong, China; christopherlai@twc.edu.hk

**Keywords:** ^1^H NMR, quercetin, adipogenesis, 3T3-L1 cells, mitochondrial membrane potential, ROS, lipid metabolism

## Abstract

(1) Background: Adipocyte differentiation involves coordinated changes in mitochondrial activity, redox balance, and cellular metabolism. Here, we examined how quercetin affects lipid accumulation and associated mitochondrial-redox and metabolic features during 3T3-L1 adipogenic differentiation by combining functional assays with time-resolved ^1^H NMR metabolomics. (2) Materials and methods: 3T3-L1 cells were differentiated for six days in the absence or presence of 20 μM quercetin. Lipid accumulation, mitochondrial membrane potential, intracellular reactive oxygen species (ROS), and intracellular metabolite profiles were assessed at defined stages of differentiation. (3) Results: Quercetin reduced lipid accumulation, mitochondrial membrane potential, and ROS accumulation, particularly at the mid-to-late stages of adipogenic differentiation. ^1^H NMR analysis showed lower lipid-associated resonances and changes in metabolites related to energy metabolism, choline metabolism, and amino acid metabolism in quercetin-treated cells. Orthogonal partial least squares-discriminant analysis (OPLS-DA) showed separation of the metabolic profiles of untreated and quercetin-treated cells at each measured stage. The variables contributing to this separation included lipid-associated resonances, lactate, 3-hydroxybutyrate, choline-related metabolites, and selected amino acids. (4) Conclusions: The results indicate that quercetin reduces lipid accumulation during adipogenic differentiation in association with changes in mitochondrial-redox status and intracellular metabolic profiles. Overall, the findings support stage-dependent alterations of metabolic profiles in quercetin-treated 3T3-L1 cells during adipogenic differentiation.

## 1. Introduction

Obesity is closely linked to type 2 diabetes mellitus, cardiovascular disease, and metabolic syndrome [1,2,3]. Expansion of adipose tissue reflects both adipocyte hypertrophy and hyperplasia, together with increased lipid deposition as preadipocytes differentiate into mature adipocytes [2,4,5]. Adipogenesis is commonly described as a transcriptionally regulated process, and peroxisome proliferator-activated receptor gamma (PPARγ) is a central regulator of lipid accumulation and adipocyte maturation [4,5]. Nevertheless, gene regulation alone does not explain the full cellular transition. Lipid synthesis, energy demand, mitochondrial activity, and redox signaling all change during differentiation, indicating that metabolism also contributes to adipocyte development [6,7].

Mitochondria support this process by supplying ATP, biosynthetic intermediates, and reactive oxygen species (ROS). A moderate rise in ROS has been reported during adipocyte differentiation and appears to participate in adipogenic signaling [8,9]. Excessive oxidative stress, however, can disturb mitochondrial activity and cellular homeostasis [2]. Thus, adipocyte maturation likely requires mitochondrial activity and redox signaling to remain within a workable physiological range.

Adipogenesis is accompanied by marked metabolic changes. The most obvious is the accumulation of triglycerides and lipid droplets [6,10]. Lactate reflects changes in glycolytic activity [11,12], whereas ketone body-related metabolites such as 3-hydroxybutyrate provide information on energy metabolism and cellular signaling [13,14]. Choline-containing metabolites are also relevant because membrane turnover and lipid droplet biogenesis increase as cells enlarge [15,16]. These metabolic features can therefore serve as useful readouts of adipocyte differentiation.

Quercetin is a dietary flavonoid present in many fruits and vegetables. It has antioxidant and anti-inflammatory properties and has also been linked to metabolic regulation [9,12]. Previous work has shown that quercetin can reduce adipogenesis and influence pathways such as AMPK, MAPK, and AKT [17,18,19,20,21]. Quercetin has also been associated with mitochondrial activity and ROS generation [22]. What remains less clear is how the metabolic profile changes over time during quercetin-treated adipogenesis.

Proton nuclear magnetic resonance spectroscopy (^1^H NMR) is well suited to follow such changes because it is reproducible, requires limited sample preparation, and detects several classes of metabolites in the same sample [23,24,25]. When combined with multivariate analysis, ^1^H NMR metabolomics can compare metabolic patterns during cell differentiation and treatment [23,24,26,27,28,29]. For adipogenesis studies, this approach is useful because lipid-associated signals, energy-related metabolites, amino acids, and membrane-associated compounds can be monitored across time [30,31,32,33,34,35].

In the present work, we examined the effects of quercetin on lipid accumulation and associated mitochondrial-redox and metabolic features during 3T3-L1 adipogenic differentiation using functional assays and time-resolved ^1^H NMR metabolomics. Cells were differentiated for six days with or without 20 μM quercetin [19]. Lipid accumulation, mitochondrial membrane potential, intracellular ROS, and metabolite profiles were measured at different stages of differentiation [8,10,36]. The temporal relationship between mitochondrial-redox changes and metabolic alterations during quercetin exposure remains unclear. Therefore, this study focused on examining stage-dependent mitochondrial-redox and metabolic alterations associated with quercetin treatment during adipogenic differentiation.

## 2. Materials and Methods

### 2.1. Experimental Design

Mouse embryo-derived 3T3-L1 preadipocytes were cultured at 37 °C in a humidified 5% CO_2_ incubator in DMEM supplemented with high glucose, sodium pyruvate, FBS, and penicillin-streptomycin [19,37,38]. Cells were passaged twice weekly and maintained in exponential growth. To assess the dose- and time-dependent effects of quercetin on cell viability, monolayers were exposed to 0, 10, 20, 40, 100, 200, or 400 µM quercetin [19,39]. Viable cells were counted directly at 0, 24, 48, and 72 h after treatment.

For differentiation experiments, 3T3-L1 preadipocytes were induced for 6 days in the absence or presence of 20 µM quercetin [18,19]. Medium was replaced every 2 days, and cells were collected for measurement of ROS and mitochondrial membrane potential (ΔΨm) and for ^1^H NMR metabolite analysis [8,23,28,36]. Adipogenesis-associated lipid accumulation was evaluated by Oil Red O (ORO) staining and lipid-associated ^1^H NMR measurements [6,10,19]. The adipogenic stock solutions contained insulin (1.5 mM), 3-isobutyl-1-methylxanthine (IBMX; 1 M), and dexamethasone (Dex; 1 mM) [5,19,37]. Aliquots were stored at −20 °C. The experiments were performed independently three times.

### 2.2. Determination of 3T3-L1 Preadipocyte Viability by Trypan Blue Staining

Cells were seeded at 3 × 10^4^ cells per well in 12-well plates and incubated with quercetin (0, 10, 20, 40, 100, 200, or 400 μM) in 1 mL of culture medium [19,39]. At 0, 24, 48, and 72 h, cells were mixed 1:1 with trypan blue and counted under a Nikon ECLIPSE ELWD 0.3/OD75 microscope Nikon Corporation, Tokyo, Japan.

### 2.3. Cell Culture and Adipogenic Differentiation

3T3-L1 cells were seeded in T25 flasks at 2 × 10^5^ cells per flask [9,19,37]. Flasks were assigned to two groups: adipogenic differentiation without quercetin and adipogenic differentiation with 20 μM quercetin [18,19]. At 80–90% confluence, the growth medium was replaced with induction medium containing 1.5 μM insulin, 0.5 mM IBMX, and 1 μM Dex; this time point was defined as Day 0 [19,37]. Medium was changed every 2 days. On Days 2 and 4, the induction medium was replaced with normal growth medium and maintained until Day 6 [37]. Cells were collected on Days 0, 2, 4, and 6 for morphology, ^1^H NMR, ROS, and mitochondrial membrane potential (ΔΨm) analyses [8,23,28,36,40].

### 2.4. Assessment of Intracellular Lipid Droplets by Oil Red O (ORO) Staining

On Days 0, 2, 4, and 6, cells were washed twice with phosphate-buffered saline (PBS), fixed with 500 μL of 4% formaldehyde for 30 min at room temperature, and rinsed three times with PBS (pH 7.4). The cells were then treated with 500 μL of 60% isopropanol in deionized water, stained with 0.3% ORO in isopropanol for 30 min, and briefly rinsed in 60% isopropanol. After three final washes with PBS, cell morphology and lipid accumulation were examined using a Nikon ECLIPSE ELWD 0.3/OD75 microscope. Images were collected from at least five fields per sample [10]. The archived experimental records do not contain sufficient detail to reconstruct the exact image-analysis software, thresholding procedure, field-selection algorithm, or blinding status used in the original relative mean red-intensity analysis; therefore, these items are reported as a methodological limitation rather than inferred retrospectively.

### 2.5. Determination of the Mitochondrial Membrane Potential (ΔΨm)

Cells were washed twice with PBS on Days 0, 2, 4, and 6, pelleted at 5000 rpm for 20 s, and resuspended in 500 µL of PBS. Mitochondrial membrane potential (ΔΨm) was assessed with Rhodamine B (product no. 83689, Sigma-Aldrich, St. Louis, MO, USA) as a fluorescent cationic probe. Cells were incubated with 1 µM Rhodamine B for 5 min, and fluorescence was measured using a CytoFLEX flow cytometer (Beckman Coulter, Brea, CA, USA) with excitation by the 488-nm laser and acquisition using the FITC-A parameter. We acquired approximately 10,000 events per sample and analyzed them using FlowJo software, v10.0.8. Cellular debris was excluded based on forward scatter (FSC) and side scatter (SSC), followed by sequential singlet gating to exclude cell aggregates and doublets. Rhodamine B fluorescence was quantified as the mean fluorescence intensity of the final gated single-cell population and used as a relative indicator of ΔΨm. Higher fluorescence was interpreted as greater potential-dependent dye accumulation, whereas lower fluorescence was interpreted as reduced dye accumulation consistent with mitochondrial depolarization [36].

### 2.6. Determination of Intracellular Reactive Oxygen Species (ROS)

Intracellular ROS was measured using the DCFH-DA assay. On Days 0, 2, 4, and 6, cells were incubated with 5 µL of 1 mM 2′,7′-dichlorodihydrofluorescein diacetate (DCFH-DA) prepared in dimethyl sulfoxide (DMSO). After 30 min at 37 °C in 5% CO_2_, cells were washed twice with PBS, harvested, centrifuged at 5000 rpm for 20 s, and resuspended in 500 µL of PBS. Fluorescence from oxidized DCFH-DA was measured using the CytoFLEX platform (A00-1-1102, Brea, CA, USA) [8].

### 2.7. Determination of Metabolites in 3T3-L1 Cell Pellet Samples by Proton Nuclear Magnetic Resonance Spectroscopy (^1^H NMR)

After the indicated incubation period, we removed the culture medium, washed the cells twice with PBS, and harvested them by trypsinization. We suspended the cells in 500 µL PBS, transferred approximately 2 × 10^6^ cells from each group to 1.5 mL tubes, and centrifuged at 7000 rpm for 1 min. The supernatant was discarded, and the prepared cell pellets were immediately processed for 1H NMR analysis without freezing or metabolite extraction. The pellets were washed with 100 µL D_2_O, centrifuged, and the supernatant was removed. Subsequently, 600 µL D_2_O containing 0.03% (*w*/*v*) TMSP-d4 was added, and the samples were vortexed for 30 s before transfer to 5-mm-diameter NMR tubes for spectral acquisition. All experimental groups were processed identically, and the resulting spectra were used for relative metabolic profiling rather than absolute quantification of extracted intracellular metabolites [28,40,41]. No separate chemical metabolic-quenching step was used in the final protocol, and the pH of individual NMR samples was not independently adjusted or documented in the archived experimental records. These factors are therefore acknowledged as limitations when interpreting the relative metabolic profiles.

**Figure 1:**
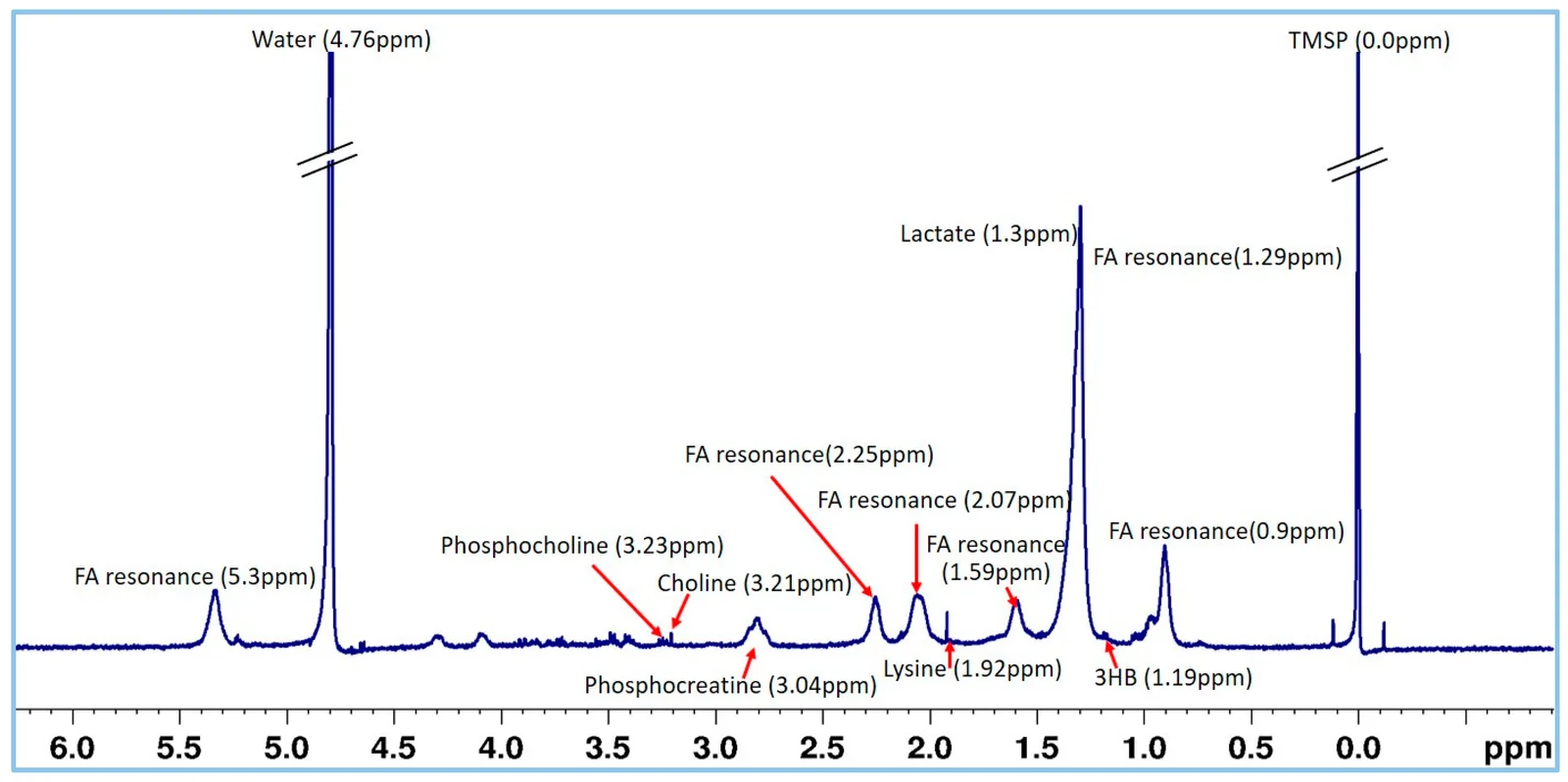
Representative ^1^H NMR spectrum of 3T3-L1 cell samples acquired on a Bruker Avance 500 MHz spectrometer (Bruker, Bremen, Germany). Peak labels indicate characteristic chemical shifts in ppm. “FA resonance” denotes a fatty-acyl-associated resonance and does not imply triglyceride specificity.

Samples were transferred to 5-mm NMR tubes, and 1D ^1^H NMR spectra were acquired on a Bruker AVANCE 500 MHz spectrometer using a CPMG-presaturation pulse sequence (cpmgpr1d) for water suppression. Spectra were recorded at 298 K with 16 scans and 4 dummy scans. The spectral width was 10 kHz, with 65,536 time-domain points and an acquisition time of approximately 3.28 s. The relaxation delay was 4 s. The transmitter frequency was 500.177 MHz, and the transmitter offset was approximately 4.70 ppm. Free induction decays were processed in TopSpin 4.0.7. An exponential window function with 0.3 Hz line broadening was applied before Fourier transformation. Spectra were manually phase- and baseline-corrected and referenced to TMSP-d_4_ at 0.00 ppm. The 0.50–6.00 ppm region was used for the analysis, and the residual water region at 4.60–5.00 ppm was excluded before integration and multivariate analysis. Spectral intensities were normalized to the total spectral area after water-region exclusion. Metabolite assignments were tentative and were based on characteristic chemical shifts, peak patterns, multiplicity information where applicable, published ^1^H NMR metabolomics literature, and spectral databases such as the Human Metabolome Database (HMDB; https://hmdb.ca) [23,28,40]. Baseline and phase corrections were performed after transformation of the free induction decay (FID) signal to the frequency domain (Figure 1) [23].

### 2.8. Statistical Analysis

Data distribution was assessed using the Kolmogorov–Smirnov and Shapiro–Wilk tests. Group differences were analyzed by two-way ANOVA with treatment and differentiation stage as the main factors, followed by Tukey’s multiple-comparisons test where appropriate to control the family-wise error rate. Data are reported as mean ± SD, and *p* < 0.05 was considered statistically significant. Given the small number of independent biological replicates (*n* = 3), normality-test results and inferential statistics were interpreted cautiously. Complete replicate-level numerical source data for some archived quantitative figure-level analyses could not be reconstructed with sufficient confidence; accordingly, those inferential results are treated as archival analyses and interpreted cautiously rather than retrospectively recalculated from inferred values. Statistical analyses were performed using IBM SPSS 17 and OriginPro 2016. Multivariate analysis was carried out in MetaboAnalyst 6.0 [23]. Metabolite peak intensities were normalized to the sum of the total spectral area and then auto-scaled to reduce the effect of differences in metabolite concentration ranges. Orthogonal partial least squares-discriminant analysis (OPLS-DA) was used to evaluate group separation and to identify variables contributing to the observed differences. Variable importance in projection (VIP) scores were interpreted together with univariate trends to avoid relying on multivariate results alone.

## 3. Results

### 3.1. 3T3-L1 Preadipocyte Cell Viability

Quercetin cytotoxicity was first evaluated in 3T3-L1 cells over a concentration range of 0–400 μM for 72 h [19,39]. At 20 μM, quercetin did not significantly reduce cell viability at 24 h compared with untreated controls. The 20 µM concentration minimized non-specific loss of viability while permitting assessment of lipid accumulation and associated metabolic and redox features during adipogenic differentiation. This concentration was therefore selected for the differentiation experiments because it allowed assessment of metabolic and redox changes without substantial loss of cell viability and was within a biologically relevant concentration range. This choice is consistent with previous reports showing that low concentrations of quercetin can affect adipocyte differentiation while maintaining cell viability [19].

### 3.2. Determination of Intracellular Lipid Droplets by Oil Red O (ORO) Staining

**Figure 2:**
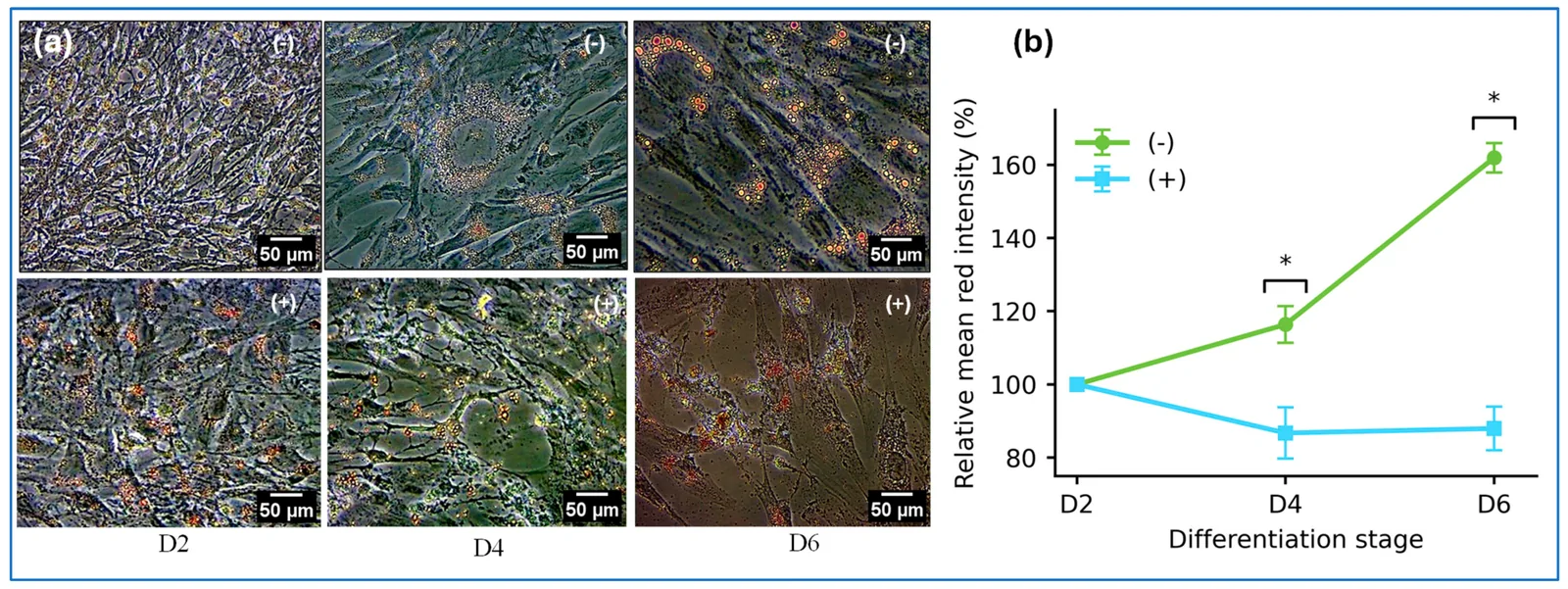
Effect of quercetin on intracellular lipid accumulation during the adipogenic differentiation of 3T3-L1 cells. (**a**) Representative Oil Red O (ORO) staining of 3T3-L1 cells differentiated in the absence (-) or presence (+) of quercetin treatment at D2, D4, and D6. Scale bar = 50 μm. (**b**) Quantitative analysis of relative mean red intensity from ORO staining. * *p* ≤ 0.05.

3T3-L1 preadipocytes were differentiated for 6 days in the absence or presence of 20 μM quercetin [18,19]. Lipid accumulation was examined by ORO staining [15]. As shown in Figure 2, lipid droplets were detected at D2, D4, and D6. In untreated cells, lipid droplets increased over time, with larger and more abundant droplets at D6, consistent with adipocyte maturation [6,10]. In quercetin-treated cultures, lipid staining remained detectable, especially at the early stage, but the number of lipid-laden cells and the staining intensity were lower at later time points. Together with the ^1^H NMR data showing reduced lipid-associated signals, these observations indicate that quercetin reduced lipid accumulation at the population level during adipogenic differentiation [18,19,28,40].

### 3.3. Determination of the Mitochondrial Membrane Potential (ΔΨm)

**Figure 3:**
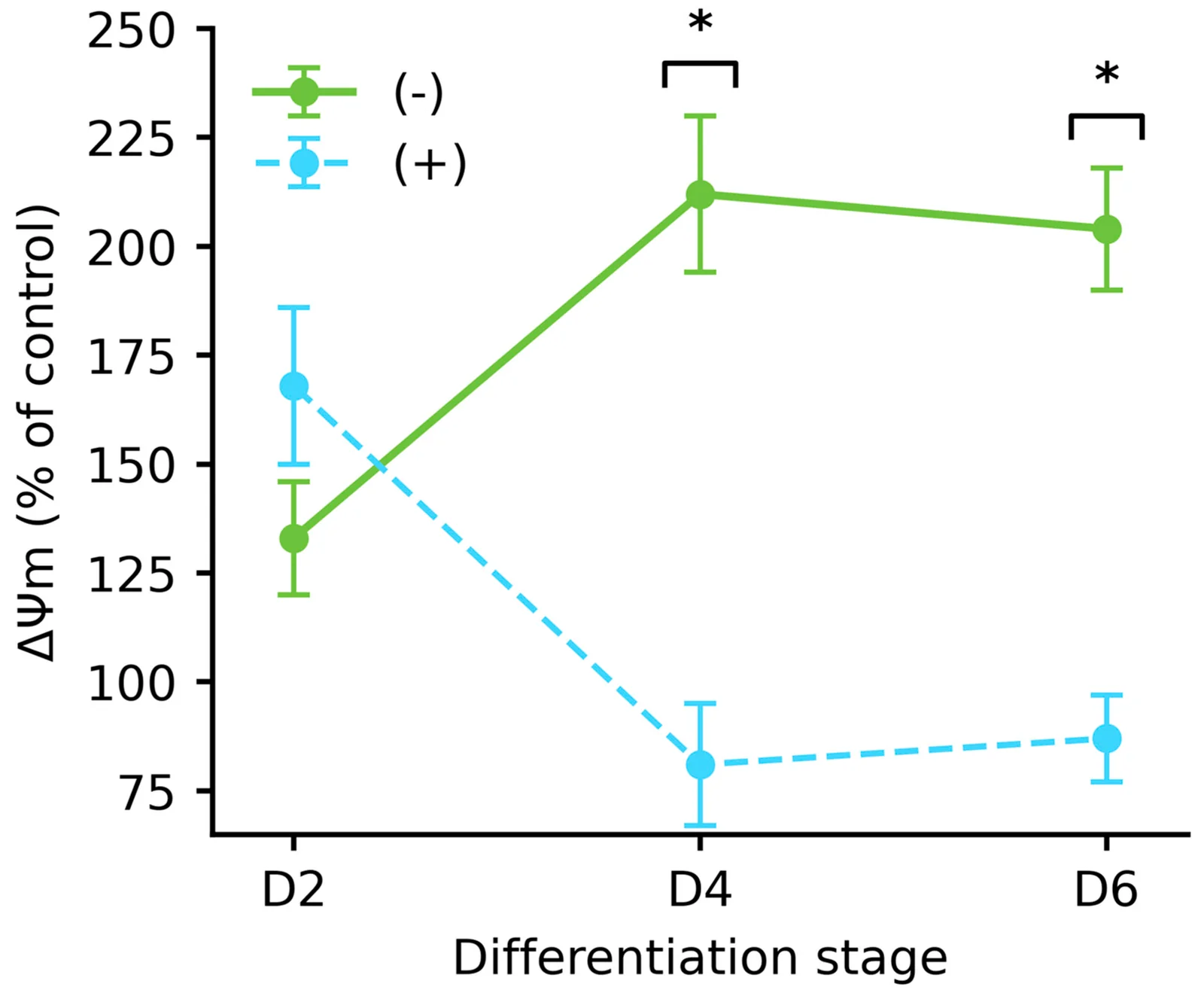
Mitochondrial membrane potential during adipogenesis. The ΔΨm of 3T3-L1 cells was monitored on Days 2, 4, and 6 after induction of differentiation in the absence (-) or presence (+) of quercetin. Data are shown as mean ± SD (*n* = 3), * *p* ≤ 0.05.

During differentiation, mitochondrial membrane potential (ΔΨm) increased in untreated cells, reaching a maximum at D4 and remaining elevated at D6 [40]. This pattern is consistent with stage-dependent changes in mitochondrial membrane polarization during adipocyte maturation [7]. Quercetin-treated cells showed a different pattern. ΔΨm was not reduced at D2 but was significantly lower at D4 and D6 than in untreated cells (* *p* < 0.05) [22,36,42]. These data indicate that quercetin treatment was associated with lower mitochondrial membrane potential during the mid-to-late stages of adipogenesis (Figure 3) [19,22,42].

### 3.4. Determination of Intracellular Reactive Oxygen Species (ROS)

**Figure 4:**
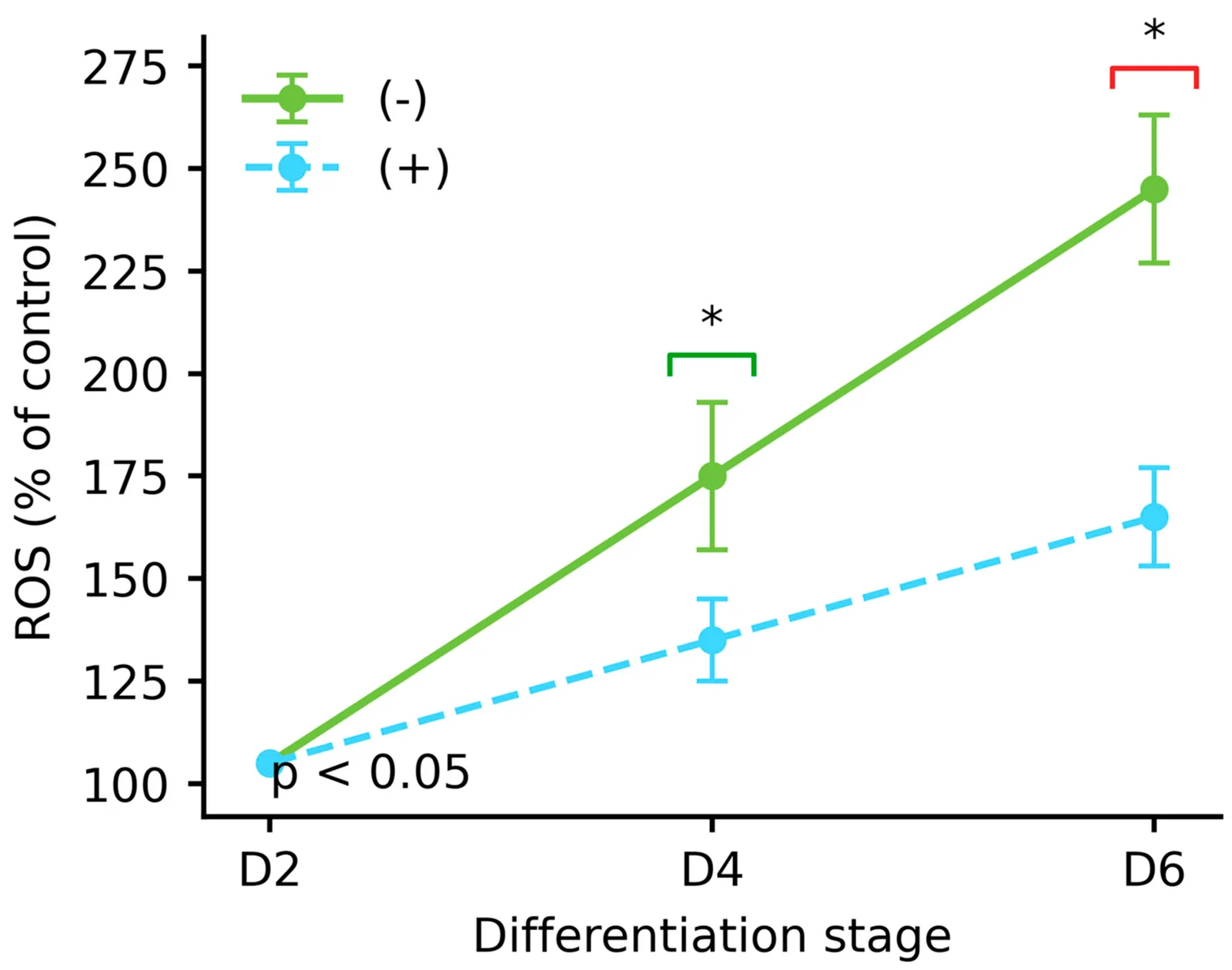
Quercetin treatment is associated with reduced ROS accumulation during adipogenesis. Intracellular ROS levels were measured using DCFH-DA fluorescence at D2, D4, and D6. In cells differentiated without quercetin (-), ROS increased during differentiation. In quercetin-treated cells (+), ROS accumulation was lower at D4 and D6. Data are shown as mean ± SD (*n* = 3), * *p* ≤ 0.05.

ROS levels increased progressively in untreated cells after D2 during adipogenic differentiation [8]. This increase is consistent with previous studies linking adipogenesis to mitochondrial activity and redox signaling [9]. In quercetin-treated cells, ROS accumulation was lower. ROS levels were comparable between groups at D2, whereas quercetin significantly reduced ROS accumulation at D4 and D6 (* *p* < 0.05) as shown in Figure 4 [8,22]. These data suggest that quercetin treatment was associated with reduced redox activation during adipogenesis. Because ROS can act as signaling molecules during adipocyte differentiation, the reduced ROS signal may reflect changes in redox-sensitive processes, although causality was not tested here [8,9].

### 3.5. Metabolic Alterations During Adipogenesis Assessed by ^1^H NMR

**Figure 5:**
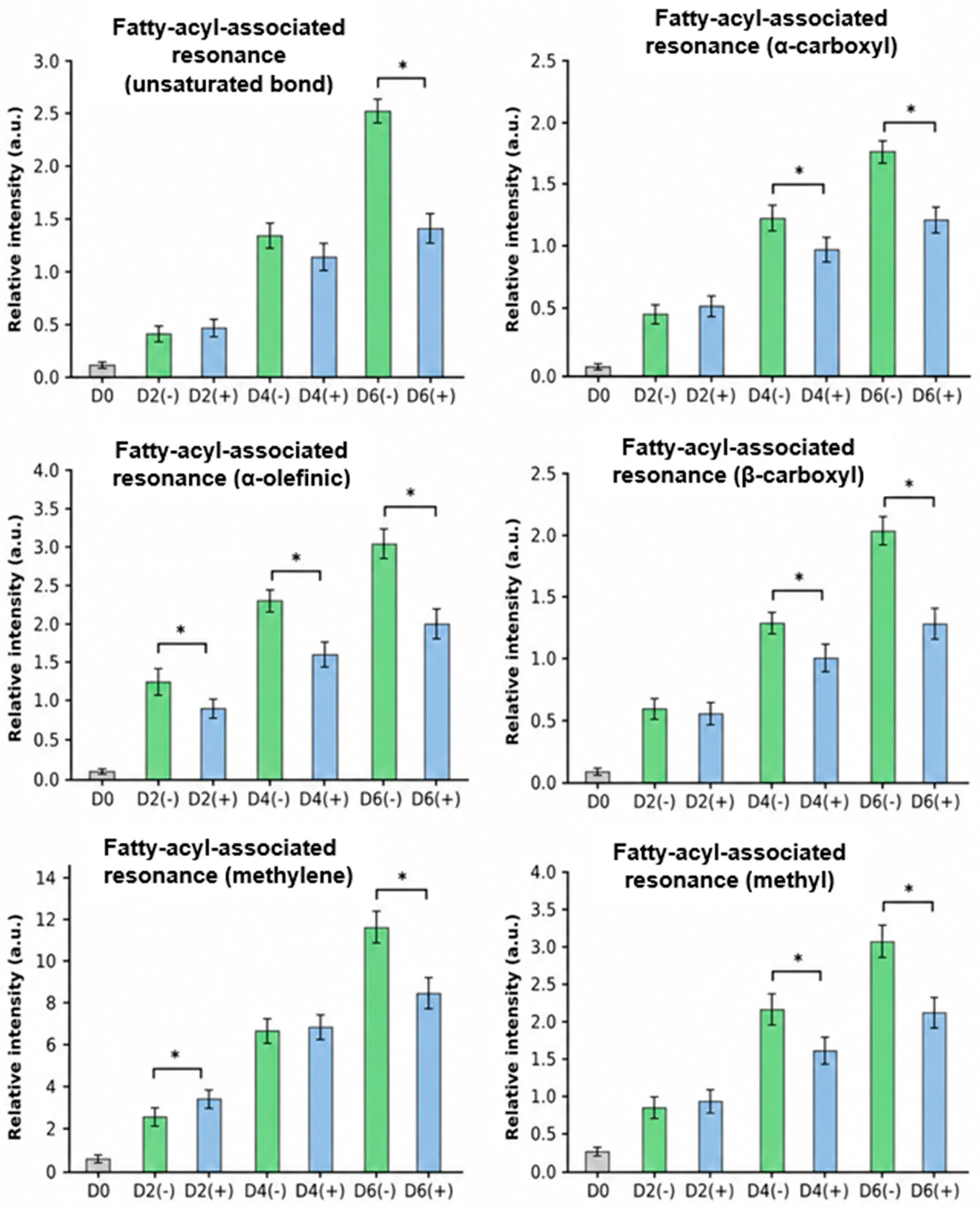
Quercetin is associated with reduced stage-dependent lipid accumulation during adipogenic differentiation in the absence (-) or presence (+) of quercetin. Lipid-associated resonances are reported according to their characteristic fatty-acyl proton environments. * *p* ≤ 0.05.

Lipid-associated ^1^H NMR resonances increased during adipogenic differentiation (Figure 5). Lipid-associated fatty-acyl resonances denoted as in Table 1 at 5.3, 2.25, 2.07, 1.59, 1.29, and 0.90 ppm increased from D2 to D6 in untreated cells [28,40,41]. These resonances correspond to unsaturated lipid protons, fatty-acyl chains, methylene groups, and terminal methyl groups, and are consistent with increased lipid storage during adipocyte maturation [6,28,41]. The pattern agrees with the ORO staining results, which showed progressive lipid droplet accumulation [6,10].

Quercetin reduced several lipid-associated resonances in a stage-dependent manner. The effect was modest at D2 and became more evident at D4 and D6. Signals at 1.29 ppm and 2.07 ppm were lower in quercetin-treated cells in the archived analysis, consistent with reduced accumulation of fatty-acyl chain-associated resonances. Because complete replicate-level numerical source data underlying the quantitative Figure 5 analysis could not be reconstructed from the archived figure-level files with sufficient confidence, the magnitude and inferential significance of these differences should be interpreted cautiously. Overall, the observed pattern is consistent with reduced lipid accumulation during the mid-to-late stages of adipogenic differentiation [18,19,36].

**Figure 6:**
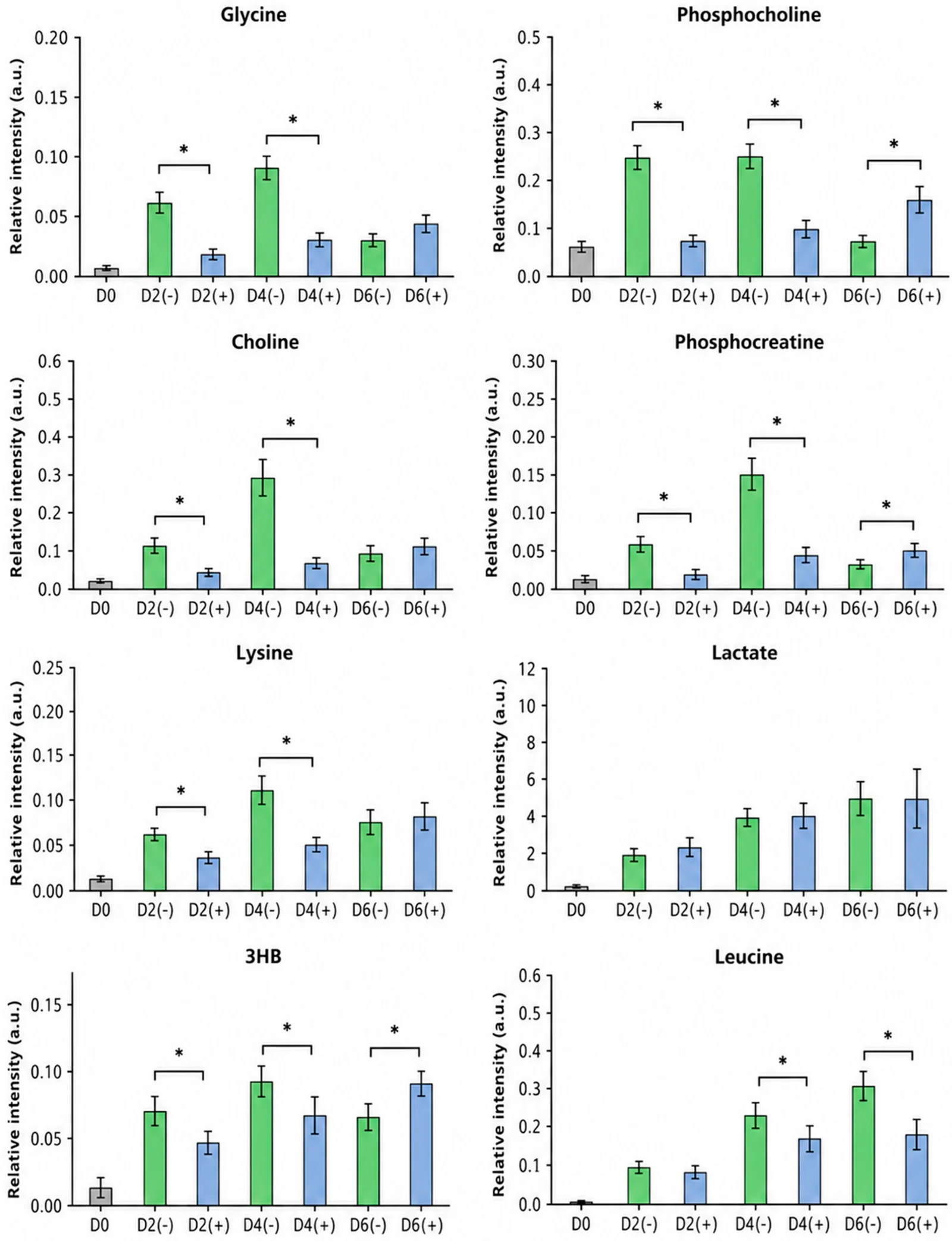
Stage-dependent non-lipid metabolic alterations during adipogenic differentiation in the absence (-) or presence (+) of quercetin treatment. 3HB (3-hydroxybutyrate). * *p* ≤ 0.05.

Non-lipid metabolites also changed during differentiation (Figure 6) [7,28,40]. In untreated cells, phosphocholine and choline, phosphocreatine, lactate, 3-hydroxybutyrate, glycine, lysine, and leucine showed stage-dependent changes [7,28,41]. Increased lactate is consistent with altered glycolytic activity, whereas changes in 3-hydroxybutyrate are consistent with changes in ketone body-related metabolism [11,12,13,14]. Variation in leucine may reflect changes in nutrient-sensitive metabolism during adipocyte maturation [43,44]. Changes in choline and phosphocholine are also compatible with altered membrane turnover during cell growth and lipid droplet formation [15,16].

Quercetin-treated cells showed changes in several non-lipid resonances, including lactate, 3-hydroxybutyrate, leucine, choline, and phosphocholine [11,12,13,14,15,16,17,20,21,43,44]. These changes were most evident during the later stages of differentiation. Taken together, the ^1^H NMR data indicate that quercetin treatment was associated with lower lipid-associated signals and shifts in metabolites linked to energy, choline, and amino acid metabolism during adipogenesis [18,19,21,28,40].

Bar graphs show relative intensities (a.u.) of lipid-associated resonances detected by ^1^H NMR at 5.3, 2.25, 2.07, 1.59, 1.29, and 0.9 ppm in control cells (D0, gray), cells differentiated without quercetin (−, green), and cells differentiated with quercetin (+, blue) at D2, D4, and D6. Lipid signals increased during differentiation in untreated cells. In quercetin-treated cells, several lipid-associated signals were lower, particularly at D4 and D6. Brackets and asterisks reproduce the original archived comparisons between (−) and (+) groups at the same stage. Data are shown as mean ± SD (*n* = 3). Because complete replicate-level numerical source data could not be reconstructed from the archived figure-level files with sufficient confidence, the inferential statistics shown in this figure should be interpreted cautiously.

The relative intensities of selected non-lipid resonances, including glycine, phosphocholine, choline, phosphocreatine, lysine, lactate, 3-hydroxybutyrate, and leucine, were quantified by ^1^H NMR in control cells (D0) and differentiated cells at D2, D4, and D6 in the absence (−) or presence (+) of quercetin. Data are shown as mean ± SD (*n* = 3). Asterisks reproduce the original archived statistical comparisons. Because complete replicate-level numerical source data could not be reconstructed from the archived figure-level files with sufficient confidence, the inferential statistics shown in this figure should be interpreted cautiously.

### 3.6. Fold Change Analysis of Metabolite Variations

Fold-change analysis was used to summarize stage-dependent changes in metabolites associated with quercetin treatment (Table 2). Values were calculated from ^1^H NMR metabolite-associated resonances in quercetin-treated cells relative to untreated cells at each stage [23,28,40]. Most lipid-associated resonances, including CH=CH (5.34 ppm), -CH_2_-CH_2_-COO (2.25 and 1.59 ppm), -CH_2_-CH=CH- (2.07 ppm), and -CH_3_ (0.90 ppm), were lower in quercetin-treated cells (fold change < 1), consistent with reduced lipid accumulation [6,28,41]. Lactate (1.33 ppm) and 3-hydroxybutyrate (1.19 ppm) also changed with treatment, suggesting differences in energy-related metabolism [11,12,13,14]. Choline and phosphocholine showed stage-dependent changes, consistent with altered membrane-associated metabolism [15,16]. Leucine, lysine, and glycine also varied across stages [44,45]. Overall, the fold-change data indicate that quercetin treatment was associated with shifts in lipid-, energy-, choline-, and amino acid-related metabolite profiles during adipogenesis [17,18,19,20,21,28,40].

### 3.7. Proposed Model of Quercetin-Associated Reduction in Lipid Accumulation During Adipogenic Differentiation

**Figure 7:**
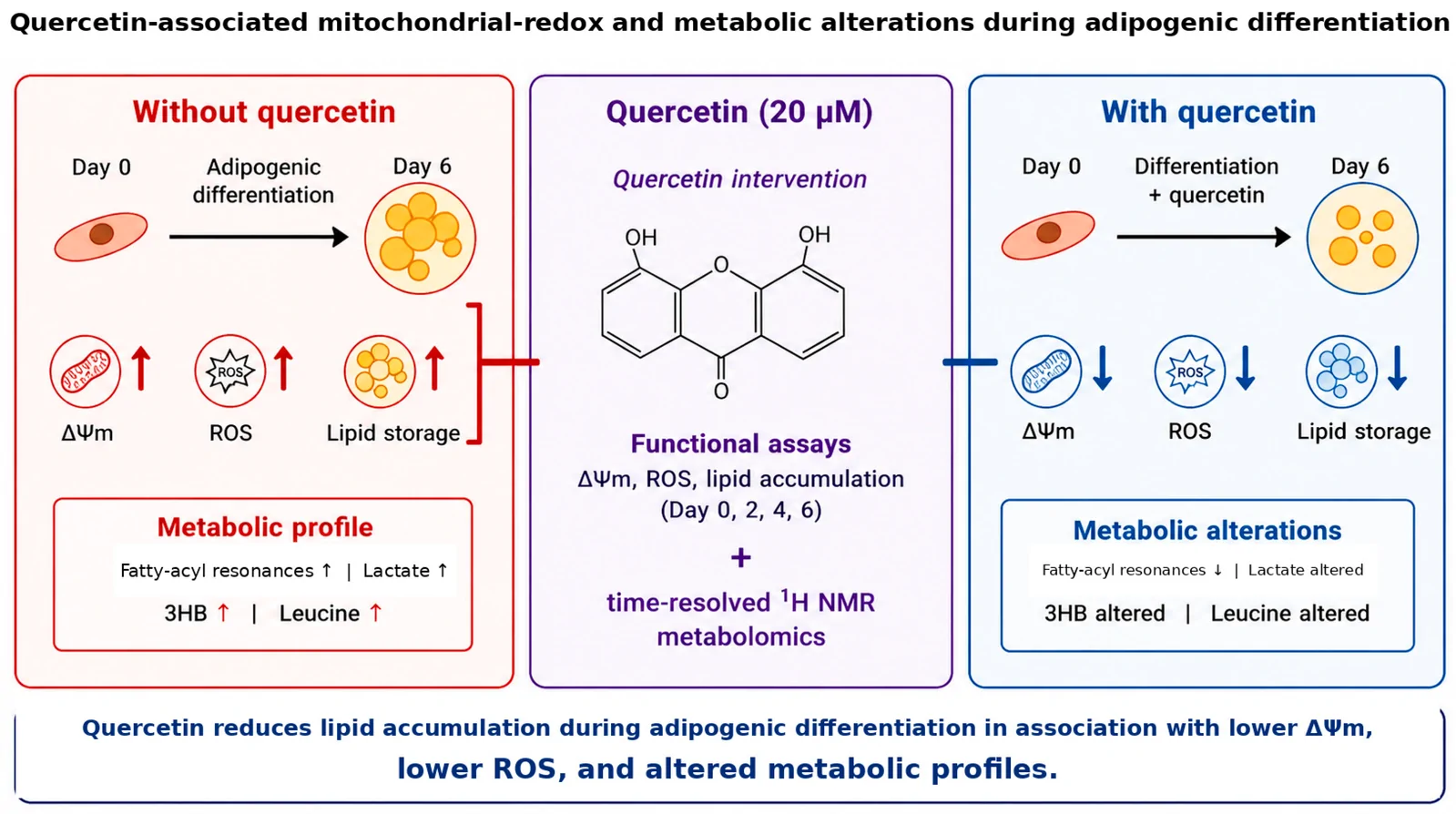
Quercetin reduces lipid accumulation and is linked to changes in metabolic processes and mitochondrial-redox status during adipogenic differentiation of 3T3-L1 cells (D0-D6). The figure compares conditions with (+) and without (−) quercetin treatment. The proposed model shows that adipogenic differentiation is associated with increased mitochondrial membrane potential (ΔΨm), ROS accumulation, and lipid storage. Quercetin treatment was associated with lower mitochondrial membrane potential, reduced ROS accumulation, lower lipid accumulation, and changes in lipid-, energy-, choline-, and amino acid-associated metabolic features. The model summarizes associations observed in this study and does not establish suppression of the complete adipogenic differentiation program.

The results support a working model in which adipogenic differentiation is accompanied by changes in mitochondrial-redox status and metabolite profiles, while quercetin treatment is associated with reduced lipid accumulation and shifts in several of these measured features [7,19,22]. The metabolomic profile (Figure 7) showed stage-dependent increases from D2 to D6 in lipid-associated resonances and selected non-lipid metabolites [7,28,40]. These changes are consistent with lipid storage, glycolytic activity, ketone body-related metabolism, and nutrient-sensitive metabolism during adipocyte maturation [6,11,12,13,14,43,44]. In quercetin-treated cells, several metabolite-associated signals were lower or shifted in pattern [17,18,19,20,21]. Adipogenic differentiation was also accompanied by increased ΔΨm and ROS accumulation [8,36]. Because mitochondrial respiration and ATP production were not measured, these observations should be interpreted as changes in mitochondrial-redox status rather than direct evidence of a specific mitochondrial mechanism. Quercetin treatment was associated with lower ΔΨm and ROS accumulation [22,46].

### 3.8. Multivariate Analysis of ^1^H NMR Metabolic Profiles

**Figure 8:**
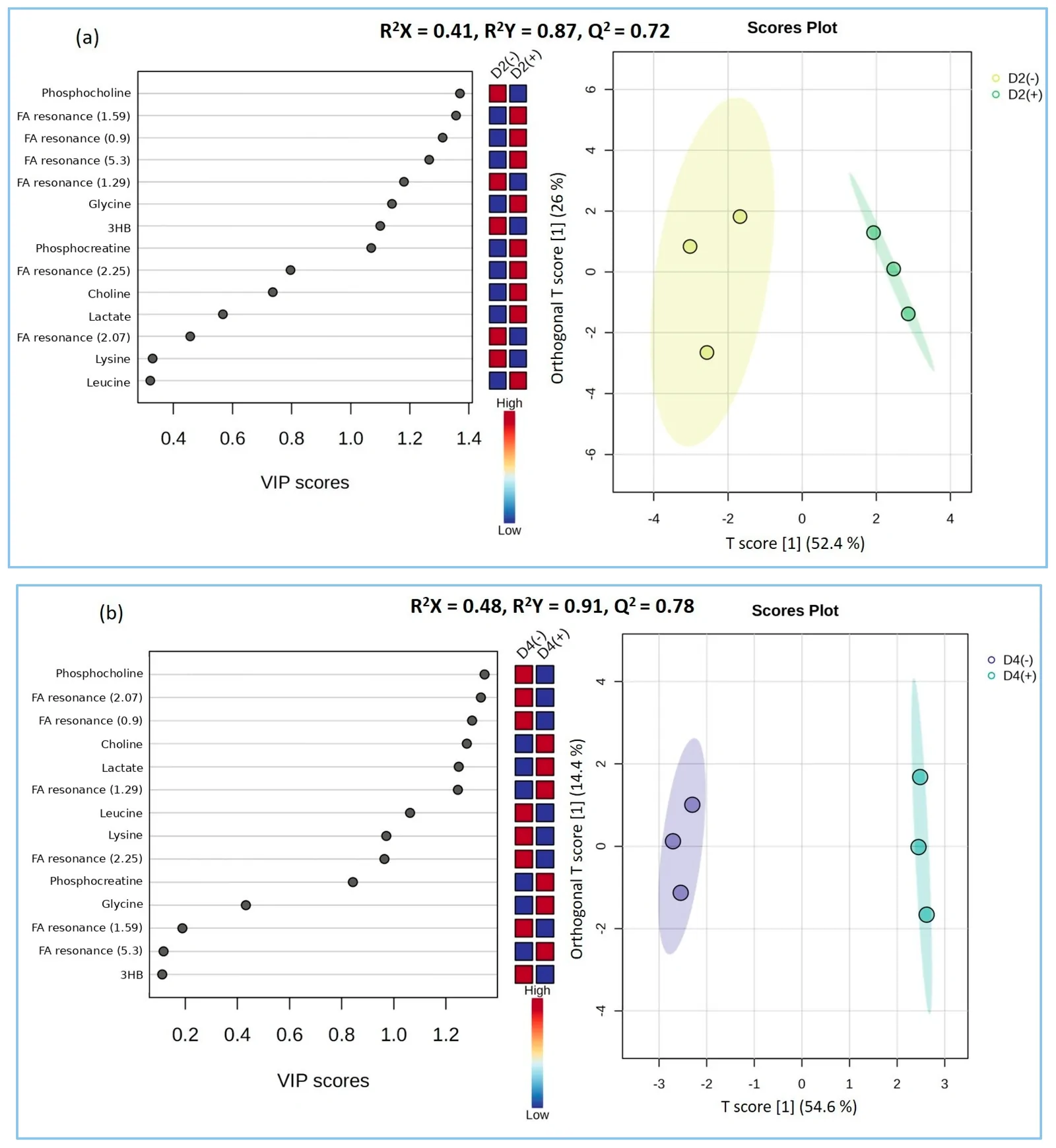

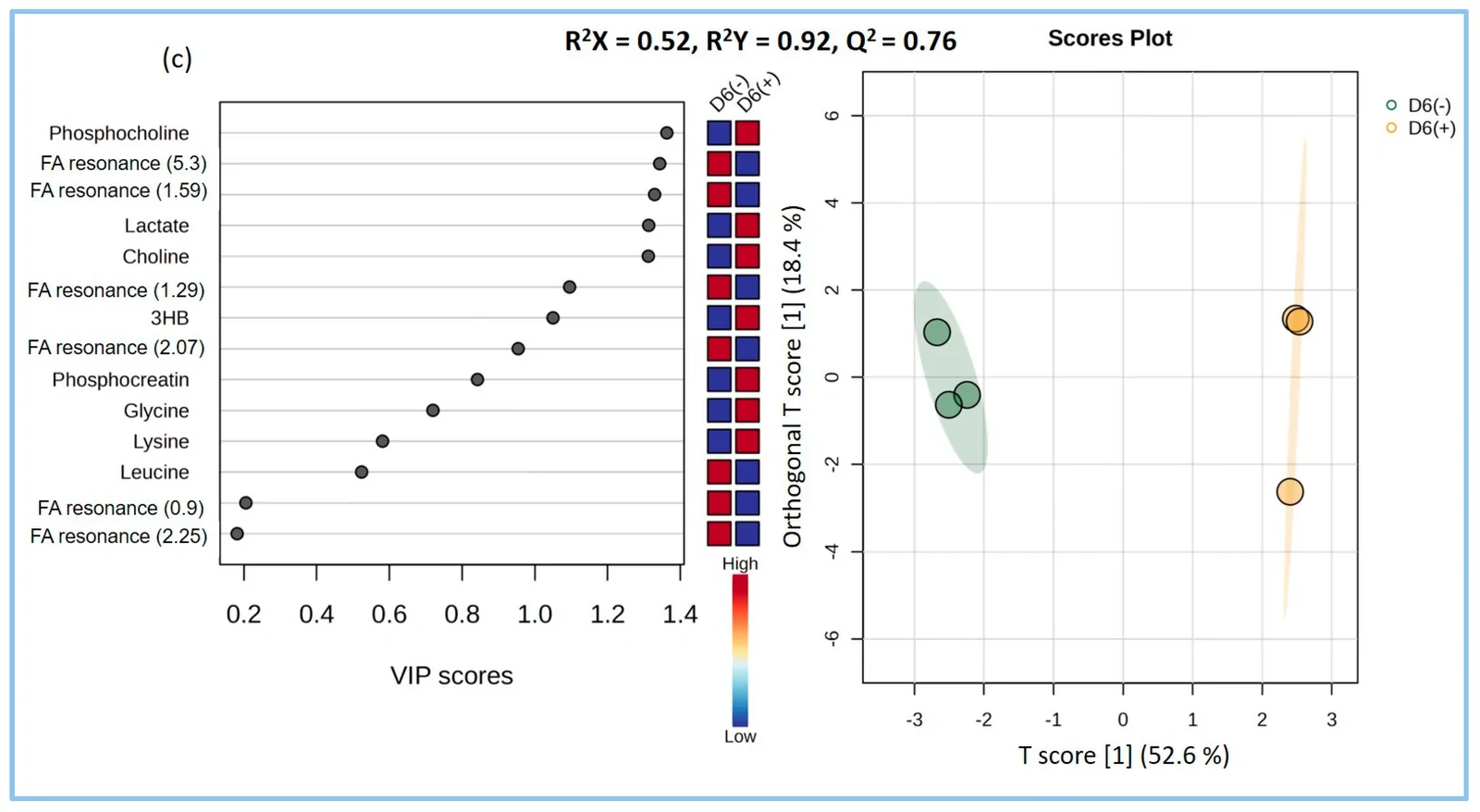
OPLS-DA analysis of metabolic profile alterations during adipogenic differentiation and quercetin treatment. (**a**–**c**) OPLS-DA models comparing cells differentiated without quercetin (-) and with quercetin treatment (+) at D2 (**a**), D4 (**b**), and D6 (**c**). VIP score plots are shown on the left, and score plots are shown on the right. Discriminating variables included fatty-acyl-associated resonances at 5.3, 2.25, 2.07, 1.59, 1.29, and 0.9 ppm, as well as phosphocholine, choline, lactate, and 3-hydroxybutyrate. In the VIP plots, labels of the form “Fatty-acyl resonance (ppm)” denote fatty-acyl-associated resonances and do not imply triglyceride specificity.

Preprocessed ^1^H NMR spectral data were analyzed by orthogonal partial least squares-discriminant analysis (OPLS-DA) to compare metabolic profiles between untreated and quercetin-treated cells. At D2, D4, and D6, the score plots showed separation between the two groups (Figure 8). The separation became more evident at later stages, indicating more pronounced differences in metabolic profiles between untreated and quercetin-treated cells as adipogenesis progressed [18,19,21].

The variable importance in projection (VIP) analysis identified lipid-associated resonances, particularly fatty-acyl-associated signals at 5.3, 2.25, 2.07, 1.59, 1.29, and 0.9 ppm, as major contributors to group separation [28,40,41]. Lactate, 3-hydroxybutyrate, choline, phosphocholine, leucine, lysine, and glycine also contributed to the models [11,12,13,14,16,17,43,44,45,47,48,49,50]. These variables were generally more influential at D4 and D6, consistent with stronger metabolic changes during adipocyte maturation. OPLS-DA was performed using normalized metabolite intensities followed by auto-scaling, consistent with the preprocessing described in the Statistical Analysis section. Model quality was assessed using the R^2^X, R^2^Y, and Q^2^ values. VIP scores were interpreted alongside univariate results to limit overinterpretation [7,28,40]. Together, the OPLS-DA and univariate analyses indicate that quercetin-treated cells had lower lipid-associated resonances and altered energy- and amino acid-related metabolite profiles [17,18,19,20,21].

## 4. Discussion

Adipogenic differentiation is usually discussed in terms of transcriptional regulation, but the differentiation process also involves metabolic, mitochondrial, and redox changes [4,5,7,42]. In this study, functional assays and time-resolved ^1^H NMR metabolomics were used to follow 3T3-L1 adipogenic differentiation in the absence or presence of quercetin. Untreated differentiation was marked by lipid accumulation, increased ΔΨm, increased ROS accumulation, and changes in both lipid-associated and non-lipid-associated NMR signals. Quercetin reduced lipid accumulation and several associated mitochondrial-redox and metabolic changes. These findings support an effect on lipid accumulation during adipogenic differentiation but do not establish suppression of the complete adipogenic differentiation program.

The clearest metabolic feature of adipogenic differentiation was the accumulation of lipid-associated resonances. Lipid-associated ^1^H NMR resonances increased from D2 to D6 in untreated cells, matching the formation and enlargement of lipid droplets observed by ORO staining. This pattern is consistent with lipid droplet biogenesis and increasing lipid storage during adipocyte maturation [6,8]. In quercetin-treated cells, lipid-associated signals were lower, especially at D4 and D6. These observations support reduced lipid accumulation during the active maturation phase. Because independent molecular markers of the adipogenic differentiation program were not measured, the data do not establish that quercetin suppressed adipocyte differentiation itself.

Mitochondrial-redox readouts changed in parallel with adipogenesis. Untreated cells showed increases in ΔΨm and intracellular ROS, particularly at D4 and D6. These findings are consistent with the increased energetic and biosynthetic requirements of differentiating adipocytes [7]. ROS has been reported to participate in adipogenic signaling, although excessive ROS can disrupt cellular homeostasis [8,9]. In our study, quercetin-treated cells showed lower ΔΨm and ROS accumulation. Because mitochondrial respiration, ATP production, mitochondrial mass, and source-specific ROS assays were not performed, the data should be interpreted as altered mitochondrial-redox status, not as proof of a defined mitochondrial mechanism.

The ^1^H NMR data also showed changes beyond lipid resonances. In untreated cells, lactate and 3-hydroxybutyrate increased during differentiation, consistent with shifts in glycolytic and ketone body-related metabolism [11,12,13,14]. Leucine varied across differentiation and may reflect changes in nutrient-sensitive metabolism during adipocyte maturation [43,44]. Choline and phosphocholine changes suggest involvement of membrane-associated metabolism [15,16]. Quercetin-treated cells showed altered resonances for several of these metabolites. Thus, the reduction in lipid accumulation associated with quercetin treatment was accompanied by selective metabolic changes rather than a uniform reduction in all measured signals.

OPLS-DA supported the presence of different metabolic profiles between untreated and quercetin-treated cells. Group separation was observed at D2, D4, and D6, indicating that quercetin treatment was associated with metabolic differences throughout differentiation. VIP analysis identified lipid-associated resonances, phosphocholine, choline, lactate, 3-hydroxybutyrate, leucine, lysine, and glycine as important variables. These metabolites are linked to lipid storage, energy metabolism, membrane turnover, and amino acid metabolism. However, the OPLS-DA results are best interpreted as pattern recognition from metabolite-associated resonances; they do not provide direct evidence of metabolic flux.

We therefore present Figure 7 as a working model. In untreated cells, adipogenesis was accompanied by increased mitochondrial polarization, ROS accumulation, and lipid-associated metabolic changes. Quercetin-treated cells showed lower ΔΨm, reduced ROS accumulation, reduced lipid accumulation, and shifts in lipid-, energy-, choline-, and amino acid-associated resonances. This model summarizes the observed associations and provides a basis for future mechanistic work rather than a causal pathway demonstrated by the present experiments.

This study has several limitations. First, the ^1^H NMR assignments were tentative and were based on 1D spectra and were not confirmed by spiking, 2D NMR, authentic standards, or LC-MS. Second, metabolic flux was not measured directly, and isotope tracing would be required to test pathway activity. Third, only one quercetin concentration (20 μM) was used for the functional and metabolomic analyses. Fourth, the experiments were repeated independently three times, which is a limited sample size for multivariate metabolomics. Fifth, complete replicate-level numerical source data underlying some quantitative figure-level analyses, including Figure 5 and Figure 6, could not be reconstructed from the archived figure-level files with sufficient confidence. Consequently, these quantitative estimates and inferential statistics cannot be independently reverified at the individual-replicate level and should be interpreted cautiously; no retrospective values were inferred or fabricated. Sixth, detailed archival information on the Oil Red O image-analysis workflow, including the exact software, thresholding procedure, field-selection algorithm, and blinding status, was not available for retrospective reporting. Seventh, the final NMR sample-preparation workflow did not include a separate chemical metabolic-quenching step, and individual sample pH was not independently documented. Finally, the work was performed in 3T3-L1 cells, and independent molecular markers of the complete adipogenic differentiation program were not measured. Therefore, the present findings support reduced lipid accumulation during adipogenic differentiation rather than suppression of adipocyte differentiation itself, and the relevance of these findings to adipose tissue in vivo requires further study. Future work combining validated metabolite identification, isotope tracing, adipogenic molecular markers, and mitochondrial functional assays would help define the mechanisms underlying quercetin-associated changes during adipogenic differentiation.

In summary, 3T3-L1 adipogenic differentiation was accompanied by lipid accumulation, increased ΔΨm, ROS accumulation, and changes in intracellular metabolite profiles. Quercetin treatment was associated with lower lipid accumulation, together with changes in several mitochondrial-redox and metabolic features, in a stage-dependent manner. The data support an association between quercetin treatment, reduced lipid accumulation, mitochondrial-redox changes, and altered metabolic profiles during adipogenic differentiation. These results may inform future studies of the metabolic regulation of adipocyte differentiation and the role of quercetin in obesity-related metabolic research.

## 5. Conclusions

This study shows that adipogenic differentiation of 3T3-L1 cells is accompanied by lipid accumulation, increased mitochondrial membrane potential (ΔΨm), ROS accumulation, and changes in intracellular metabolite profiles. Quercetin reduced lipid accumulation and several associated changes, particularly during the mid-to-late stages of differentiation. Time-resolved ^1^H NMR metabolomics showed lower lipid-associated resonances and alterations in metabolites related to energy, choline, and amino acid metabolism in quercetin-treated cells. Overall, quercetin reduced lipid accumulation during adipogenic differentiation in association with mitochondrial-redox and metabolic alterations. These findings do not establish suppression of the complete adipogenic differentiation program. Metabolic flux analysis, mitochondrial respiration assays, validated adipogenic molecular markers, and metabolite validation will be needed to clarify the underlying causal mechanisms.

## Figures and Tables

**Table 1:** List of identified fatty-acyl-chain-associated resonances and their chemical shifts in the ^1^H NMR spectrum of 3T3-L1 cells [35].

No.	Chemical Shift (ppm)	Type	Hydrogen Atom Position (Red Color)
1	5.34	Unsaturated bond	-CH=CH
2	2.25	α-Carboxyl	-CH_2_-CH_2_-COO
3	2.07	α-olefinic	-CH_2_-CH=CH-
4	1.59	β-Carboxyl	-CH_2_-CH_2_-COO
5	1.29	Methylene	-(CH_2_)n-
6	0.90	Methyl	-CH_2_-CH_3_

**Table 2:** Fold change in metabolite levels following quercetin treatment during adipogenesis.

No.	Metabolites	Chemical Shift (ppm)	Fold Change (+)/(−)		
			D2	D4	D6
1	Fatty-acyl-associated resonance (unsaturated bond)	5.34	1.12	0.86	0.56
2	Glycine	3.56	0.36	0.30	1.56
3	Phosphocholine	3.23	0.28	0.32	2.00
4	Choline	3.21	0.34	0.22	1.20
5	Phosphocreatine	3.04	0.31	0.28	1.57
6	Fatty-acyl-associated resonance (α-carboxyl)	2.25	1.23	0.81	0.71
7	Fatty-acyl-associated resonance (α-olefinic)	2.07	0.74	0.66	0.67
8	Lysine	1.92	0.56	0.39	1.05
9	Fatty-acyl-associated resonance (β-carboxyl)	1.59	0.97	0.79	0.64
10	Lactate	1.33	1.25	1.05	1.01
11	Fatty-acyl-associated resonance (methylene)	1.29	1.51	1.03	0.67
12	3-Hydroxybutyrate	1.19	0.73	0.73	1.35
13	Leucine	0.98	0.91	0.75	0.63
14	Fatty-acyl-associated resonance (methyl)	0.90	1.10	0.72	0.70

## Data Availability

The original contributions presented in this study are included in the article. Further inquiries can be directed to the corresponding authors.
